# Acute physicians in medical leadership: Essential, or nice to have?

**DOI:** 10.1016/j.fhj.2026.100535

**Published:** 2026-06-25

**Authors:** Clare Carasco, Sanjay Krishnamoorthy

**Affiliations:** West Middlesex Hospital, London, United Kingdom

**Keywords:** Acute physician, Leadership, National Health Service

## Abstract

According to Charles Darwin’s theory of natural selection, it is not necessarily the strongest or most intelligent organism that survives, but the one most responsive to change. The ability to adapt has previously been one of the core strengths of the National Health Service (NHS) and the healthcare professionals that work within it. ‘Reform or die’ was the choice for the NHS described in the ʻ10 Year Health Plan’. Therefore, we must evolve; to secure our survival, we must engage with leadership. It can no longer be a tick-box on the journey to Certificate of Completion of Training (CCT). If we want to influence the direction of healthcare, we must have a seat at the table. Diagnosing and treating illness is only a fraction of what clinicians of the future will do; we will need to develop into clinical leaders working throughout the system. A change of this magnitude will inevitably take time, but it is now that a strategic choice needs to be made to invest in our workforce and leadership training. And, arguably, who better to lead the way than the acute physician?

## Introduction

According to Charles Darwin’s theory of natural selection; it is not necessarily the strongest or most intelligent organism that survives, but the one most responsive to change. The ability to adapt has previously been one of the core strengths of the National Health Service (NHS) and the healthcare professionals that work within it. Arguably, none more so than the acute physicians.

**Fig. 1:**
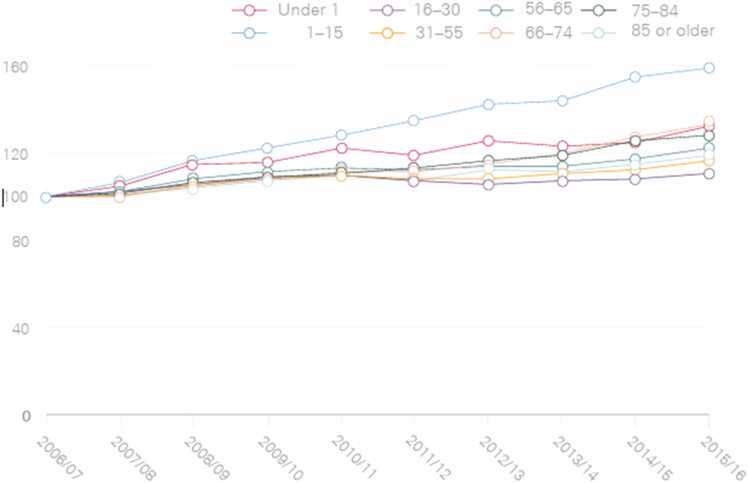
Relative change in the number of emergency admissions per year (index 2006/07=100)*: Reproduced with permission from Emergency admissions by age group (England),^2^*The Health Foundation, 2018, analysis of Hospital Episodes Statistics data,*www.health.org.uk/reports-and-analysis/briefings/emergency-hospital-admissions-in-england.

**Fig. 2:**
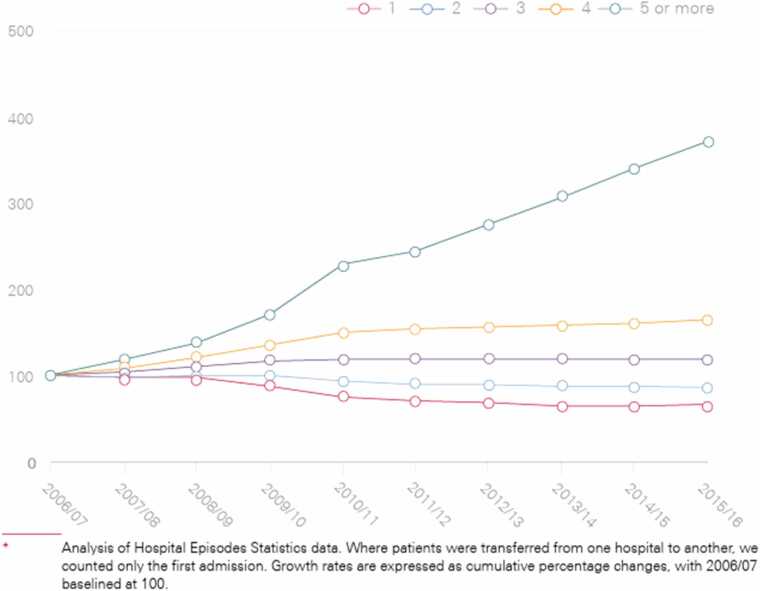
Relative change in the number of emergency admissions per year (index 2006/07=100)*: Reproduced with permission from Emergency admissions by number of health conditions (England),^2^*The Health Foundation, 2018, analysis of Hospital Episodes Statistics data,*www.health.org.uk/reports-and-analysis/briefings/emergency-hospital-admissions-in-england.

Acute medicine as a specialty was born through that need to change, through the development of a new pattern of working, creation of acute medical units, and ultimately establishing itself as a subspecialty training programme in 2003.^1^ The phenotype of the population presenting acutely in hospital has changed. Gone are the days of ‘one patient, one problem’, swiftly replaced by the new multimorbid, socially challenging patient. Medical admissions are increasing in complexity – one in three admitted in 2015/16 had five or more health conditions, compared with one in ten between 2005/6. Concurrently, the number of patients aged 85 and over being admitted to acute medical beds has grown at a greater rate than any other age group over the past decade (Fig. 1, Fig. 2).^2^

Demand has increased rapidly, with a 42% increase in emergency admissions in England between 2006 and 2018,^3^ far outstripping supply, with the number of hospital beds falling from 299,000 in 1988 to 141,000 in 2020.^4^ This, combined with chronic reductions in real-time funding, workforce crisis, and external factors such as COVID-19 and Brexit, has meant that the NHS is struggling. In order for our NHS to survive and strive to its status of world-class healthcare, transformative change is necessary and perhaps acute physicians are perfectly poised to lead this change.

## Why acute physicians?

We begin by considering the issue of increasing demand on secondary care services fuelled by an aging and increasingly comorbid population.^5^ Queuing theory tells us that systems are most efficient when they operate at 85% capacity.^6^ However, our hospitals often run at bed occupancy rates between 95% and 99%,^7^ leading to increasing inefficiency as a result of a significant demand–supply disequilibrium.

Therefore, optimisation of patient flow through the hospital is of the utmost importance. Lord Darzi’s independent investigation of the NHS in England stated that improvement in operational management was critical in order to achieve this.^8^

Hospital operational management involves the planning, coordination and control of daily clinical and non-clinical activity to ensure safe, efficient and high-quality care. Familiarity with the hospital pathways, timely consultant-led decision making and recognition of need for specialist input allows acute physicians to have both control and understanding of the determinants of patient flow. Therefore, when working at almost maximum capacity, their unique skillset and position of influence are not only valuable, but essential to ensure that operational efficiency is achieved without compromising safety.

However, optimising flow within the hospital alone is only part of the solution. It requires thinking outside of the box; quite literally outside of the four walls of the hospital. A cornerstone of the ʻ10 Year Health Plan’ is to shift care from the hospital to the community.^9^ True improvement in patient flow requires reduction in traditional fragmented care and movement towards patients moving smoothly between the components of the integrated care system.

While simple in idea, the reality is such that this shift in healthcare delivery will be anything but. Integrated care systems are complex ecosystems of partners with competing interests and differing financial pathways, often bound by levels of bureaucracy. Acute physician training aims to equip physicians with a variety of transferrable skills such as strategic thinking, prioritisation and negotiation to operate effectively in complex and uncertain environments such as these.

**Fig. 3:**
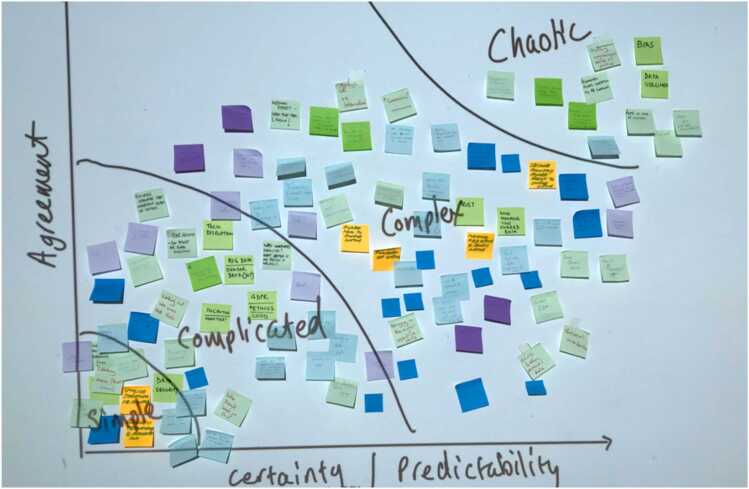
The Stacey Matrix; an example in practice.^11^*Open Data Institute, 2018, use of the Stacey Matrix as part of a Department for Transport – Open Data Institute workshop on data sharing opportunities and challenges in the aviation sector,*https://assets.publishing.service.gov.uk/media/5c0fe542ed915d0b7268ee71/dft-odi-aviation-data-discovery-report.pdf.

As a result, acute physicians are often extremely skilled at managing risk and making fast-paced challenging decisions. The Stacey Matrix,^10^ a framework for exploring difficulty in decision making dependent on degree of agreement and certainty, would place acute physicians working most often between the complicated and complexity zones (Fig. 3). According to theory, working in this area is only possible with effective collaboration, a critical quality for leaders and one that acute physicians demonstrate daily. For example, a complex discharge requires coordination between multiple healthcare professionals inside and outside of the hospital, the patient and family to ensure a safe, timely and appropriate exit from the hospital.

It seems a reasonable assumption that acute physicians trained in managing complexity on the acute floor and daily multidisciplinary collaboration might be especially well suited to senior leadership roles managing multi-faceted problem-solving alongside multiple stakeholders. Utilisation of this skillset and engagement with system partners has the potential to co-create innovative services that can be designed around and delivered in settings appropriate for population needs at a system level.

The first step towards making the progression from clinician to clinical leader a possibility is to challenge the perception of the acute physician as a ‘jack of all trades’ calling upon other specialties for advice. Arguably, it is the inherent humility of the acute physicians to recognise that they need help from others that represents one of their greatest strengths. Leadership expert Simon Sinek is clear that humility should not be misinterpreted as lack of confidence of understanding, but being open to the ideas of others.^12^ This is further supported by the work of Daniel Goleman, an American psychologist, who popularised the concept of emotional intelligence being as crucial for success as intelligence quotient.^13^ A significant component of emotional intelligence is self-awareness and therefore the ability to understand yourself and what is to be gained by involving others to achieve the desired goal. It has been demonstrated repeatedly in a variety of settings that combining insights and ideas can drive innovation.^14^

We will move on to consider the practicalities of how clinicians become leaders. In 2011, a King’s Fund report stated that one of the ‘defining weaknesses of the NHS over the decades has been the lack of involvement of clinicians in management’.^15^ For too long, an ‘us vs them’ mentality has hindered progress. In addition, even for those clinicians with an interest, traditionally leadership training has lacked structure and consistency, and had limited availability below consultant level. Positively, there appears to be a change in the tide with increasing attention towards the development of clinical leaders, with an understanding that while the process is likely to be both complex and time-intensive, it will be a fruitful investment.^9^

Acute medicine is one of the first specialties to actively encourage and provide dedicated time for the development of leadership skills as a ‘specialty skill’ during training. This positive relationship with leadership development can be seen in the growing Royal College of Physicians Chief Registrar Acute Medicine cohorts; a leadership development programme for resident doctors where time is split between clinical and leadership. This protected time allows clinicians the freedom to learn and creates an environment where the skills nurtured are seen equally as valuable as their clinical skills.

Furthermore, pragmatically a consultant acute physician holds a unique position in relation to time. While seen at times as always ‘on call’, they are not restricted by vast clinic administrative burdens of specialty colleagues or target pressures of emergency department colleagues. The career path is such that it allows space to cultivate learning in both operational and strategic domains of leadership.

**Fig. 4:**
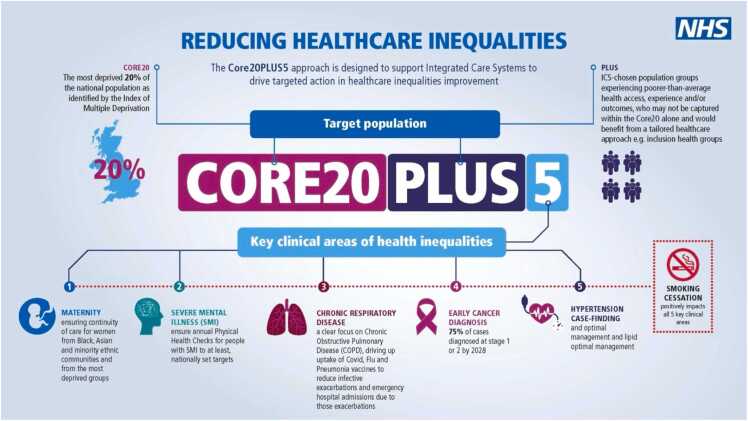
National Health Service Core20Plus5 Infographic,^17^*National Health Service England, 2023, Core20Plus5 approach to health inequalities,*https://www.england.nhs.uk/about/equality/equality-hub/national-healthcare-inequalities-improvement-programme/core20plus5/.

Involvement in healthcare strategy, from national to local level, is of particular importance in relation to health inequality. Health inequality is defined as avoidable, unfair and systematic differences in health between different groups of people. In recent years health inequalities have widened further in England, with the difference in life expectancy between the most and least deprived 10% of the population being up to 10 years and a reduction in healthy years lived.^16^ While the majority of health inequalities are a result of differences in social determinants of health, within healthcare settings we have the opportunity to minimise inequality. The Core20plus5 is a framework developed to help systems ensure that resources are utilised to provide fairer outcomes for all (Fig. 4).^17^ Often the patients that are at the highest risk of experiencing inequality have minimal engagement with healthcare, hence the opportunity to intervene and support these groups is limited. Acute physicians, alongside emergency department colleagues, are the most likely to interact with and gain a greater understanding of these groups and how to support them. It is therefore essential that they are engaged with the strategic direction of the hospital and the integrated care system to ensure that future services are developed and existing services are adapted to ensure that they do not become inequitable while striving for efficiency.

## Conclusion

‘Reform or die’ was the choice for the NHS described in the ʻ10 Year Health Plan’.^9^ As per Charles Darwin, we must evolve and engage with leadership; both as the NHS and as healthcare professionals. It can no longer be a tick-box on the journey to Certificate of Completion of Training (CCT). If we want to influence the direction of healthcare, we must have a seat at the table. Diagnosing and treating illness is only a fraction of what clinicians of the future will do; we will need to develop into clinical leaders working throughout the system.

Acute physician training is already headed in the right direction, but has the potential for even greater advancement. If we consider the model of learning at medical school: the initial years are focused on scientific theory, followed by an apprenticeship-style learning to integrate theoretical knowledge into clinical practice. This model aims to equip doctors with the knowledge, skills and attitudes required for safe patient care that are continuously honed and developed throughout their careers. We suggest that leadership training for acute physicians should follow a similar model; education, experience and ongoing development.

Formally incorporating leadership education into the acute physician training curricula would provide an essential foundation of the theory. By understanding the hospital ecosystem and how it interacts within the wider healthcare environment, acute physicians, often naturally skilled at prompt pragmatic decision-making, have the potential to develop a more strategic approach to decision-making with both the patient and population level in mind.

However, theory alone we well know will often not result in meaningful change. This requires experiential learning. Dedicated time spent throughout training alongside senior leaders would allow the opportunity for mentorship and real-world application of theory under supervision. In an ideal world, experience at a hospital, ICS and national level would provide much-needed insight into the current and future challenges to face the NHS.

Nurturing lifelong learning in leadership for the acute physician requires a culture that will support it. Challenging the status quo in the role of a physician will not be easy and there will inevitably be resistance to change. Creation of leadership networks to connect individuals with a passion for leadership would provide a safe space to share ideas, discuss challenges and further learning, as well as creating a community of pioneering physicians well-adapted to the changing NHS.

A change of this magnitude will inevitably take time, money and a significant change in mind-set for many, but it is now that the strategic choice needs to be made to invest in our acute physician workforce to develop a cohort of leaders who have the ability to lead the transformation of our NHS into one that is truly fit for the future.

## CRediT authorship contribution statement

**Clare Carasco:** Writing – review & editing, Writing – original draft, Visualization, Resources, Project administration, Data curation, Conceptualization. **Sanjay Krishnamoorthy:** Writing – review & editing, Supervision, Resources, Data curation Conceptualization.

## Funding

This research did not receive any specific grant from funding agencies in thepublic, commercial or not-for-profit sectors

## Declaration of Competing Interest

The authors declare that they have no known competing financial interests or personal relationships that could have appeared to influence the work reported in this paper.

## References

[1] Dowdle R. Society of Acute Medicine UK. History of Society of Acute Medicine. Available from: 〈https://www.acutemedicine.org.uk/our-history〉 Accessed 4th February 2026.

[2] Steventon A. (2018). Briefing: Emergency Hospital Admission in England: Which May Be Avoidable and How?.

[3] Deeny S. (2018). Briefing: Reducing Emergency Admissions: Unlocking the Potential of People to Better Manage Their Long-Term Conditions.

[4] Ewbank L. Report: NHS hospital bed numbers. Policy, finance and performance; 2021. Available from: 〈https://www.kingsfund.org.uk/insight-and-analysis/long-reads/nhs-hospital-bed-numbers〉 Accessed 4th February 2026.

[5] Office of National Statistics UK. Population estimates. Available from: 〈https://www.ons.gov.uk/peoplepopulationandcommunity/populationandmigration/populationestimates〉 Accessed 4th February 2026.

[6] Erlang A. (1909). The theory of probabilities and telephone conversations. Nyt Tidsskr Mat B.

[7] Campbell D. (2017). NHS Bosses Sound Alarm over Hospitals Already Running at 99% Capacity.

[8] Darzi A. Report: independant investigation of the National Health Service in England; 2024. Available from: chrome-extension://efaidnbmnnnibpcajpcglclefindmkaj/〈https://assets.publishing.service.gov.uk/media/66f42ae630536cb92748271f/Lord-Darzi-Independent-Investigation-of-the-National-Health-Service-in-England-Updated-25-September.pdf〉 Accessed 4th February 2026.

[9] Starmer K, Streeting W, DHSE. Report: 10 year Health Plan for England; fit for the future; 2025. Available from: chrome-extension://efaidnbmnnnibpcajpcglclefindmkaj/〈https://assets.publishing.service.gov.uk/media/6888a0b1a11f859994409147/fit-for-the-future-10-year-health-plan-for-england.pdf〉 Accessed 4th February 2026.

[10] Stacey R. (2011). Strategic Management and Organizational Dynamics: The Challenge of Complexity.

[11] ODI. Open Data Institute: data sharing opportunities and challenges in the aviation sector; 2018. Available from: chrome-extension://efaidnbmnnnibpcajpcglclefindmkaj/〈https://assets.publishing.service.gov.uk/media/5c0fe542ed915d0b7268ee71/dft-odi-aviation-data-discovery-report.pdf〉 Accessed 11th March 2026.

[12] Sinek S. (2017). Leaders Eat Last.

[13] Goleman D. (1995). Emotional Intelligence: Why It Can Matter More Than IQ.

[14] Hansen M., Birkenshaw J. (2007). The innovation value chain. Harv Bus Rev.

[15] The Kings Fund report. The future of leadership and management in the NHS; 2011. Available from: chrome-extension://efaidnbmnnnibpcajpcglclefindmkaj/〈https://assets.kingsfund.org.uk/f/256914/x/82055428e3/future_leadership_nhs_2011.pdf〉 Accessed 4th February 2026.

[16] Jefferies D, et al. The Kings Fund report: what are health inequalities. Determinants of health; 2025. Available from: 〈https://www.kingsfund.org.uk/insight-and-analysis/long-reads/what-are-health-inequalities#:∼:text=Life%20expectancy%20is%20closely%20related,expectancy%20by%20deprivation%20have%20widened〉. Accessed 4th February 2026.

[17] NHS England. Core20plus5 (adults) – an approach to reducing healthcare inequalities. Available from: 〈https://www.england.nhs.uk/about/equality/equality-hub/national-healthcare-inequalities-improvement-programme/core20plus5/〉 Accessed 4th February 2026.

